# Editorial: The European Patients Academy on Therapeutic Innovation (EUPATI) Guidelines on Patient Involvement in Research and Development

**DOI:** 10.3389/fmed.2018.00310

**Published:** 2018-11-08

**Authors:** Per Spindler, Beatriz S. Lima

**Affiliations:** ^1^Biopeople, Faculty of Health and Medical Sciences, University of Copenhagen, Copenhagen, Denmark; ^2^Instituto de Investigação do Medicamento (iMed.ULisboa), Lisbon, Portugal

**Keywords:** EUPATI, european patients academy on therapeutic innovation, patient involvement, patient advocacy, innovative medicines

Our colleagues and we are working toward improving health by speeding up the development of and patient access to innovative medicines, particularly in areas where there is an unmet medical or social need. It appears pivotal that key players of health care research collaborate on involving patients in research, which may hugely benefit the medicines development process: by bringing their priorities, perspectives, and knowledge about their own diseases to the table, patients can contribute to developing better treatments. Thus, there is a call for action to trigger a major rethink of the way patients and the public understand the medicines development process and their own involvement. There is an additional need to shape and facilitate the ways patients and patient organizations can be involved to handle the many complex ethical, legal and other issues intrinsic to the development process.

The European Patients' Academy on Therapeutic Innovation (EUPATI) was established as part of the partnership between the European Union and the European pharmaceutical industry, the so-called Innovative Medicines Initiative (IMI). EUPATI has set up structures to develop and disseminate accessible, well-structured, comprehensive, scientifically reliable, and user-friendly educational material for patients on the processes of medicines research and development (R&D). Like EUPATI, we believe that once armed with a deeper understanding patients, patient experts and patient advocates will be even more empowered to work effectively with the relevant authorities, healthcare professionals and industry to influence the medicines development process for the benefit of patients and society.

Patient organizations, academia, not-for-profit organizations, and pharmaceutical companies are represented in the EUPATI partnership. Together, through reviews and a process of public consultation, sets of policies and standards for patient's involvement in medicines research and development have been published. Thus, through the collaboration between multiple stakeholder and interest groups, conflict of interest in presentations and views is managed and controlled through transparent procedures. We therefore envisage that the guidelines will be used in relevant stakeholder communities and that they will contribute to the development of internal operating procedures in organizations and companies.

Each of four contemporary fields of patient involvement in medicines research and development are covered in publications (Figure 1).

**Figure 1:**
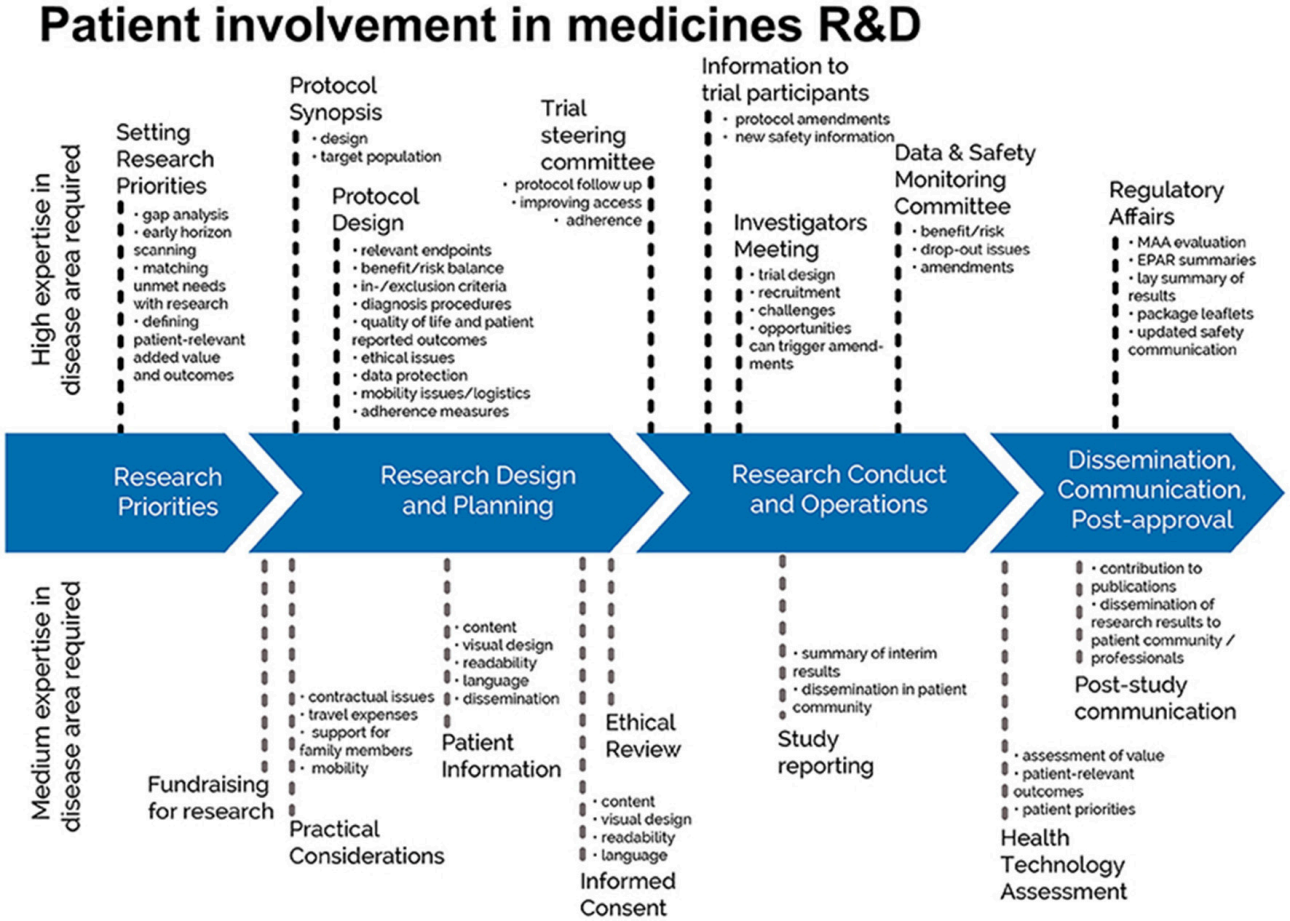
Patient involvement in medicines R&D: Patients can meaningfully contribute across the process of medicines R&D. This diagram illustrates the R&D process that require expertise in the respective disease areas. The guidances for patient involvement covered by EUPATI is the ethical review, the regulatory processes including regulatory affairs, Health Technology Assessment (HTA) and the industry-led research and development of medicines. © EUPATI, under a Creative Commons license CC BY-NC-SA 4.0. Used with permission.

First, Health Technology Assessment (HTA). HTA is to inform decision making by health care policy makers and is the basic process that evaluates the use of health technologies and generally involves a critical review of international evidence related to clinical effectiveness of the health technology vs. the best standard of care. The outcome of HTA is recommendations whether or not a health technology should be used, and if so, how it is best used and which patients are most likely to benefit from it. Thus, the patient perspectives on experiences and preferences in the HTA process are valuable. The EUPATI Guidance for Patient Involvement in Medicines Research and Development: Health Technology Assessment provides recommendations for activities to support patient involvement in HTA bodies and specific guidance for individual HTA processes (Hunter et al.). The publication also discusses the recent progress in, and continuing barriers to, patient involvement in HTA.

Secondly, the EUPATI guidance for patient involvement in medicines research and development (R&D); Guidance for pharmaceutical industry-led medicines R&D covers the interaction between patients and the pharmaceutical industry within all functions throughout the medicines R&D lifecycle in relation to medicines for human use (Warner et al.). This publication relates to activities both pre-approval and post marketing, and involving both individuals and groups of patients.

Thirdly, the EUPATI and Patients in Medicines Research and Development: Guidance for Patient Involvement in Ethical Review of Clinical Trials gives recommendations for involving patients in the work of ethics committees (Klingmann et al.). This publication provides additionally definitions and a presentation of the broader context of involvement of patients in ethical evaluations.

Finally and fourthly, the EUPATI and Patients in Medicines Research and Development: Guidance for Patient Involvement in Regulatory Processes covers patient involvement in the regulatory field and draws on the “Framework for interaction between the European Medicines Agency (EMA) and patients and consumers and their organizations” (Haerry et al.). It expands on the EMA framework, specifically including National Competent Authorities (NCAs). It sets out objectives for patient involvement in medicines regulation and recommends concrete suggested working practices. It is primarily aimed at regulatory authorities wishing to interact with patients or their organizations in their activities but could also be considered by patients/patient organizations planning to collaborate with regulatory authorities.

We are thankful to the team of collaborating organizations and individuals in EUPATI and IMI for the production of these sets of guidelines, and we are looking forward to fruitful follow-on actions aimed at providing accessible medicines to patients in need. We trust that patients' involvement is one of the significant elements of medicines research and development in the future, and we wish you a pleasant and useful read.

## Author contributions

All authors listed have made a substantial, direct and intellectual contribution to the work, and approved it for publication.

### Conflict of interest statement

The authors declare that the research was conducted in the absence of any commercial or financial relationships that could be construed as a potential conflict of interest.

